# Comparative Analysis of AI Tools for Disseminating ADA 2025 Diabetes Care Standards: Implications for Cardiovascular Physicians

**DOI:** 10.1111/1753-0407.70072

**Published:** 2025-03-06

**Authors:** Tengfei Zheng

**Affiliations:** ^1^ State Key Laboratory for Innovation and Transformation of Luobing Theory; Key Laboratory of Cardiovascular Remodeling and Function Research of MOE, NHC, CAMS and Shandong Province; Department of Cardiology Qilu Hospital of Shandong University Jinan China

**Keywords:** artificial intelligence, cardiovascular care, clinical decision support, diabetes guidelines

To the Editor,

Artificial intelligence (AI) models are increasingly used in clinical practice, including medical education and the dissemination of updated clinical guidelines. In this study, we evaluated four AI tools—ChatGPT‐4o, ChatGPT‐o1, ChatGPT‐o3Mini, and DeepSeek—to assess their ability to summarize the *Standards of Care in Diabetes—2025* from the American Diabetes Association (ADA) for cardiovascular physicians in primary care settings [1].

Using a standardized prompt, we compared the AI‐generated summaries across 10 key metrics, including accuracy (alignment with ADA 2025 guidelines), completeness (inclusion of core topics such as glycemic targets, blood pressure management, lipid control, and pharmacologic strategies), clarity (readability and conciseness for cardiovascular physicians), clinical relevance (utility for real‐world cardiovascular practice), consistency (logical coherence and uniformity in recommendations), evidence support (reference to supporting studies and ADA standards), ethics (neutral and evidence‐based recommendations), timeliness (inclusion of the latest ADA updates), actionability (practical guidance for cardiovascular physicians), and fluency (professional language and structure). Each AI tool was rated on a 0–5 scale for each category, yielding a total possible score of 50 points. All summaries were anonymized to remove identifiers. Each model (ChatGPT‐4o, ChatGPT‐o1, ChatGPT‐o3Mini, and DeepSeek) was then tasked with evaluating all four anonymized summaries, including its own output, using the predefined 10 metrics. For each model, the four scores assigned by the evaluators (including self‐evaluation) were averaged to calculate the final score per metric.

Our evaluation showed that ChatGPT‐o1 performed best (48.3/50), excelling in completeness (5.0), clinical relevance (5.0), and actionability (5.0), with comprehensive coverage of diabetes screening, cardiovascular risk assessment, hypertension/lipid management, and multidisciplinary collaboration (Table 1). However, its evidence support (4.0) required improvement. ChatGPT‐4o (45.5/50) demonstrated strengths in clarity (4.8) and structure but had limitations in timeliness (4.5) and evidence support (3.3), as it failed to incorporate 2025 guideline updates and lacked specific research references. The free models, O3Mini (47.3/50) and DeepSeek (47.3/50), performed comparably to paid tools. O3Mini excelled in consistency (5.0) and CKD/heart failure monitoring, while DeepSeek prioritized concise cardiovascular risk management (clarity: 5.0). Both free models, however, scored lower in completeness (O3Mini: 4.8; DeepSeek: 4.5) and evidence support (O3Mini: 4.0; DeepSeek: 3.8), reflecting insufficient integration of 2025 updates and trial data (Table 1).

**TABLE 1:** Cross‐Sectional analysis and strengths and weaknesses of AI tools.

Cross‐sectional analysis				
Metric	ChatGPT‐4o	ChatGPT‐o1	ChatGPT‐o3mini	DeepSeek
Accuracy	5.0	5.0	5.0	4.8
Completeness	4.0	5.0	4.8	4.5
Clarity	4.8	4.8	4.8	5.0
Clinical relevance	4.8	5.0	4.8	5.0
Consistency	4.8	5.0	5.0	5.0
Evidence support	3.3	4.0	4.0	3.8
Ethics	5.0	5.0	5.0	5.0
Timeliness	4.5	4.8	4.5	4.5
Actionability	4.5	5.0	4.8	4.8
Fluency	5.0	4.8	4.8	5.0
Total	45.5/50	48.3/50	47.3/50	47.3/50
COST	$20/month	$200/month	Open Access	Open Access

AI tool		Strengths and weakness		
ChatGPT‐4o		Strengths: ChatGPT‐4o has a clear structure with a focus on key areas such as blood pressure control, lipid management, antiplatelet therapy, SGLT2 inhibitors, and GLP‐1 receptor agonists. It is suitable for clinical doctors to read, with smooth expression and concise content. Weakness: It lacks the new changes from the 2025 guidelines, and there is no noticeable difference compared to the 2024 version. The evidence support is average, as no specific research data or guideline references are provided.		
ChatGPT‐o1		Strengths: ChatGPT‐o1 provides a comprehensive summary, covering all key points of interest to cardiovascular doctors, including diabetes screening, cardiovascular risk assessment, hypertension and lipid management, antiplatelet therapy, heart failure, and CKD management. The language is clear and the logic is rigorous, emphasizing multidisciplinary collaboration, with high practical guidance for primary care physicians. Weakness: The evidence support could be stronger, and the latest ADA research data could be referenced, such as the new clinical trial information added in the 2025 guidelines.		
ChatGPT‐o3mini		Strengths: ChatGPT‐o3mini has a well‐organized structure and smooth language, covering diabetes management, cardiovascular protection, heart failure, and CKD monitoring, making it highly valuable for clinical doctors. It emphasizes multidisciplinary collaboration and highlights the important role of cardiovascular doctors in diabetes management. Weakness:It does not fully reflect the latest content from the 2025 guidelines, primarily focusing on the core points of the 2024 version. The evidence support is slightly improved, but it still lacks references to the latest research data from 2025.		
DeepSeek		Strengths: DeepSeek has concise content, focusing on cardiovascular risk management, hypertension and lipid‐lowering strategies, and the cardiovascular protective effects of diabetes medications. It emphasizes multidisciplinary collaboration and annual updates, making it suitable for doctors to quickly grasp key points. Weakness: It lacks the latest updates from the ADA 2025 guidelines, with little reflection on the new guideline content. The evidence support is weak, as it does not mention key research data or trials.		

Among the most critical takeaways for cardiovascular physicians were the importance of individualized glycemic targets, the use of SGLT2 inhibitors and GLP‐1 receptor agonists for cardiovascular protection, and the necessity of multidisciplinary collaboration for diabetes management. However, while AI‐generated summaries provide a convenient way to access guidelines, the lack of explicit reference to primary sources remains a limitation that requires human oversight.

Given the potential for AI to support clinical decision‐making, integrating these models with validated medical sources and interactive decision‐support systems could further enhance their utility [2]. Cost considerations are notable: DeepSeek and O3Mini are open‐access, whereas ChatGPT‐4o and ChatGPT‐o1 require subscriptions. Despite its higher cost, ChatGPT‐o1 outperformed other tools, while ChatGPT‐4o lagged behind free models in total scores, raising questions about its cost‐effectiveness (Table 1). Future studies should explore the integration of AI‐generated summaries with interactive decision‐support systems to optimize patient care.

## Author Contributions

T.Z. conceived and designed the study, performed all analyses, interpreted the results, and wrote the manuscript. The author approved the final version of the manuscript and takes full responsibility for all aspects of the work.

## Disclosure

The author has nothing to report.

## Conflicts of Interest

The author declares no conflicts of interest.
